# Fabrication and Characterization of Apple-Pectin–PVA-Based Nanofibers for Improved Viability of Probiotics

**DOI:** 10.3390/foods12173194

**Published:** 2023-08-24

**Authors:** Asad Nawaz, Sana Irshad, Noman Walayat, Mohammad Rizwan Khan, Muhammad Waheed Iqbal, Xiaofang Luo

**Affiliations:** 1 Hunan Engineering Technology Research Center for Comprehensive Development and Utilization of Biomass Resources, College of Chemistry and Bioengineering, Hunan University of Science and Engineering, Yangzhou 425199, China; luoxf@huse.edu.cn; 2 Institute for Advanced Study, Shenzhen University, Shenzhen 518060, China; sanairshad55@gmail.com; 3 College of Tea Science and Tea Culture, Zhejiang Agriculture and Forestry University, Hangzhou 310007, China; noman.rai66@gmail.com; 4 Department of Chemistry, College of Science, King Saud University, Riyadh 11451, Saudi Arabia; 5 School of Food and Biological Engineering, Jiangsu University, Zhenjiang 212013, China; waheediqbal@ujs.edu.cn

**Keywords:** apple pectin, nanofibers, probiotics, electrospinning, viability

## Abstract

In the current study, apple-pectin-based novel nanofibers were fabricated by electrospinning. Polyvinyl alcohol (PVA) and apple pectin (PEC) solution were mixed to obtain an optimized ratio for the preparation of electrospun nanofibers. The obtained nanofibers were characterized for their physiochemical, mechanical and thermal properties. The nanofibers were characterized using scanning electron microscopy (SEM), Fourier transform infrared (FTIR) spectroscopy and thermogravimetric analysis (TGA). Furthermore, an assay of the in vitro viability of free and encapsulated probiotics was carried out under simulated gastrointestinal conditions. The results of TGA revealed that the PVA/PEC nanofibers had good thermal stability. The probiotics encapsulated by electrospinning showed a high survival rate as compared to free cells under simulated gastrointestinal conditions. Furthermore, encapsulated probiotics and free cells showed a 3 log (cfu/mL) and 10 log (cfu/mL) reduction, respectively, from 30 to 120 min of simulated digestion. These findings indicate that the PVA/PEC-based nanofibers have good barrier properties and could potentially be used for the improved viability of probiotics under simulated gastrointestinal conditions and in the development of functional foods.

## 1. Introduction

Probiotics, particularly *Lactobacillus acidophilus*, have gained significant attention in recent years due to their potential health benefits. These beneficial bacteria have been associated with improving gut health, enhancing the immune system and even aiding in weight management. However, the viability and stability of probiotics during storage and digestion pose significant challenges [1]. To overcome these limitations, studies have been exploring innovative strategies to enhance the viability of probiotics, such as the utilization of electrospinning techniques to fabricate nanofibers of biopolymers [2].

Electrospinning has emerged as a promising method for the production of nanofibers with high surface-to-volume ratios, uniform structures and excellent mechanical properties. By incorporating probiotics within electrospun nanofibers, their viability and stability can be significantly improved [3]. In this context, the fabrication of nanofibers using pectin and PVA offers a viable approach to enhance the viability of *L. acidophilus*.

Pectin has received a lot of attention as a desired biopolymer, with apples being one of its main sources. It is a complex polysaccharide made up of galacturonic acid and other natural sugar residues. Apple pectin, in particular, has demonstrated excellent biocompatibility and biodegradability, making it suitable for food packaging and energy storage [4]. Additionally, it possesses unique physicochemical properties, such as gel-forming ability, film-forming capability and emulsifying properties, which further contribute to its appeal as a modified (esterification) biopolymer for the encapsulation of probiotics [5].

The use of apple-based pectin in electrospinning processes offers several advantages. Firstly, pectin can form homogenized solutions, which provide a suitable environment for probiotic survival and growth. The hydrophilic nature of pectin ensures the retention of moisture, thereby preventing the dehydration of the probiotic cells [6]. Moreover, pectin has a mucoadhesive property, which enables it to adhere to intestinal mucosa and enhances the colonization of probiotics in the gut. This property is particularly important for the successful delivery of probiotics to the target site. Furthermore, apple-based pectin is considered safe for human consumption and has a low immunogenicity, which makes it a suitable material for encapsulation [7].

This study aims to assess the viability of a probiotic strain (*L. acidophilus*) by adopting the novel approach of electrospinning. In this study, nanofibers of apple pectin and PVA were fabricated and further characterized for physiochemical, structural and morphological properties. Moreover, the viability of free and encapsulated probiotic cells was assessed under thermal and simulated gastrointestinal conditions. This study will provide meaningful information about the survival of probiotics and open a new window for the development of new functional foods and drug delivery.

## 2. Materials and Methods

### 2.1. Procurement of Materials

Pectin (74% galaturonic acid, from apple peel) was obtained from Sigma-Aldrich, Shanghai, China. The polyvinyl alcohol (PVA, molecular weight = 30,000 g/mol), *Lactobacillus acidophilus*, probiotic strain and MRS (De Man, Rogosa and Sharpe) agar used in this study were purchased from Sigma-Aldrich Shanghai Co., Ltd., Shanghai, China. Other chemicals and reagents used in this study were used without further purification.

### 2.2. Bacterial Culture Activation

A bacterial culture of *L. acidophilus* was prepared by following the method of Aider-Kaci et al. [8], whereas the MRS agar method was used for the activation of lactic acid bacteria. MRS agar medium (100 mL) was prepared in distilled water and sterilized via an autoclave at 121 °C and 15 psi for 15 to 20 min. The medium was allowed to cool to 45–50 °C before pouring into sterile Petri dishes. The bacterial culture was spread evenly over the surface of the agar, and the MRS agar plates were inoculated with the bacterial culture using a sterile loop or pipette. The plates were incubated at 30–37 °C as this was an appropriate temperature to culture lactic acid bacteria. Colonies started to appear within 24 h. The cell count was recorded and the cell concentration was adjusted at 10^10^ cfu/mL.

### 2.3. Preparation of Solutions

For the fabrication of nanofibers, 10% (*w*/*v*) PVA solution was prepared by adding 10 g of PVA to 100 mL of distilled water on a magnetic stirring plate (Wisestir MSH-2OD, Miliot Science, Porvoo, Finland) at 90 °C for 2 h. Next, 10% (*w*/*v*) pectin (PEC) solution was prepared by adding 10 g of PEC to 100 mL of distilled water on a magnetic stirring plate at 80 °C for 3 h. A standard ratio of PVA/PEC blends was formulated as by Xu et al. [9] with slight modifications. Due to low electrical conductivity and high surface tension, it was difficult to electrospin the pure pectin solution as the biopolymer solution had not achieved optimal viscosity and conductivity. Therefore, PVA was used to achieve better electro-spinnability and the fabrication of continuous and uniform nanofibers. The ratio of the 20 g electrospun blend was maintained at 10:10 for PVA:PEC.

### 2.4. Fabrication PEC/PVA Nanofibers

The bacterial suspension containing *L. acidophilus* was magnetically stirred for 20 min at 36 °C to homogenize the bacterial pellets/colonies. A micropipette was used to add 1 mL of bacterial culture in the blend of PEC/PVA. Dope solution was prepared for the electrospinning process using a nanospider machine (NS 1S500U, Elmarco, Liberec XI, Czech Republic). The carriage of the nanospider was filled with PEC/PVA solution and a conductive sheet was applied for the collection of flying nanofibers. The conduction rate was set at 140 mm/s, the voltage was set at 118.8 kV and a distance of 10 cm was maintained between the carriage and collector sheet. The process of fabrication was started and the solution was completely consumed after a few hours. The foil was taken out of the machine and a sheet of nanofibers was obtained upon the collector fabric.

### 2.5. Encapsulation Efficiency

The encapsulation efficiency (%) of the PEC/PVA nanofibers loaded with probiotics, *L. acidophilus,* was determined by following the method of Xu et al. [9]. Briefly, 5 g of probiotic-loaded nanofiber was suspended in phosphate-buffered solution with pH adjusted to 7.4. Agar plates and dilutions of samples were prepared. The samples were poured onto the agar plates and incubated for 24 h at 37 °C, then the cell count was recorded. The following equation was used to calculate the encapsulation efficiency:

Encapsulation efficiency (%) = (Amount of substance encapsulated/Total amount of substance added) × 100

### 2.6. Characterization of PEC/PVA Nanofibers

#### 2.6.1. Zeta Potential (ξ)

Zeta potential is an important factor to determine the colloidal dispersion of nanofibers. The zeta potential of the PEC/PVA nanofibers was determined by the method of Liu et al. [10], via Zeta Potential Analyzer (NANOTRAC Wave II, Malvern Panlytical, Malvern, UK). Replicates were taken 10 times to obtain a mean value. The zeta potential value (ξ) was calculated using the following equation:

ξ = η μ/ε

where η = viscosity of solution, μ = electrophoretic mobility of solution and ε = dielectric coefficient of solution.

#### 2.6.2. Thickness

An electronic digital micrometer (H-2780, Uline, Detroit, MI, USA) was used to determine the thickness (nanometers) of the PEC/PVA nanofibers. The thickness was determined at various positions of the nanofibers. An average of ten replicates were taken for the mean values.

#### 2.6.3. Mechanical Properties of Nanofibers

The fabricated nanofibers of PEC/PVA were assessed for their barrier properties, such as thickness, tensile strength (TS) and elongation at break (EAB). The thickness of the nanofibers was determined using scanning electron microscopy, whereas to assess the tensile strength and EAB, the nanofibers were subjected to control stretching force until they broke. The applied force and resulting deformation were measured to calculate the TS and EAB using the following formulas:

TS: σ = F_max/A_0 EAB = (L_max − L_0)/L_0 ∗ 100

#### 2.6.4. Morphological Characterization

The blank and probiotic-loaded nanofiber mats were subjected to scanning electron microscopy, SEM (Emcraft cubeseries, Thermo Fisher, Seoul, Republic of Korea), for the morphological characterization. The diameters (±SD) of the PEC/PVA nanofiber (blank and probiotic-loaded) mats were measured at 100 different positions on an SEM image.

#### 2.6.5. Molecular Characterization

Fourier transform infrared spectroscopy (FTIR- Spectrum Two-Perkin Elmer, Waltham, MA USA) was performed for the molecular characterization of the free and probiotic-loaded PEC/PVA nanofibers. The secondary structure was assessed using OMNIC Software 8.3 (Thermo Nicolete Corp., Madison, WI, USA) by removing background peaks. A total of 32 scans were performed between 400 and 4000 cm^−1^ at room temperature.

#### 2.6.6. Thermogravimetric Analysis

To analyze the thermal behavior of the PVA powder, PEC powder and PVA/PEC nanofibers, both blank (P_0_) and probiotic-loaded (P_1_), a thermogravimetric analyzer, TGA (TGA 701, Leco, St. Joseph, MI, USA), was used.

### 2.7. In Vitro Simulated Gastrointestinal Analysis

The viability of probiotics is a critical factor in their efficacy for various applications. To assess the viability of the free and probiotic-loaded nanofibers under simulated gastrointestinal (GI) conditions, the method described by Singh et al. [11] was followed, with slight modifications. Simulated gastric fluid (SGF) and simulated intestinal fluid (SIF) were prepared in deionized water. The pH of the SGF was adjusted to 3 by adding hydrochloric acid (HCl), pepsin and sodium, whereas the pH of the SIF was adjusted to 6.8 by adding dibasic potassium phosphate and potassium hydroxide (KOH). The SGF solution was incubated for 2 h for gastric simulation under anaerobic conditions, whereas the SIF solution was incubated for 3 h under anaerobic conditions for intestinal simulation. The experiment was performed in triplicate for both treatments (free and encapsulated). Colonies were counted in terms of cfu/mL, and the results are expressed as log_10_ values.

### 2.8. Statistical Analysis

The data were statistically analyzed with an appropriate model of SPSS for Windows (version 22 IBM SPSS Statistics, IBM Corp., New York, NY, USA). The collected data were statistically analyzed through mean values and standard deviation in order to evaluate the statistical significance of every parameter [12]. The significant differences between treatments were analyzed using one-way ANOVA and Duncan’s multiple range test at a level of *p* < 0.05.

## 3. Results and Discussions

### 3.1. Encapsulation Efficiency (EE%)

Encapsulation efficiency is a critical factor in probiotic encapsulation as it influences the probiotic’s protection, viability, stability, controlled release and dose consistency. The overall quality, effectiveness and reliability of a probiotic can be enhanced by optimizing encapsulation efficiency [13]. The results of EE and loading efficiency (LE%) are shown in Table 1. The results revealed that the probiotic cells were successfully loaded in the PEC/PVA nanofibers; the EE was recorded as 82.90%. EE refers to the ability of the nanofiber matrix to effectively encapsulate and protect the probiotic cells. The solubility of the biopolymer in the solvent used for nanofiber production affects the uniformity and homogeneity of the nanofiber matrix [14]. Proper solubility ensures even distribution of probiotics within the nanofiber structure, leading to better EE. The mechanical properties of biopolymers influence the structural integrity and stability of the nanofiber matrix. However, a strong and flexible biopolymer helps to maintain the structural integrity during encapsulation and protects the probiotics from external stresses, such as shear forces [15]. The successful loading of probiotic cells has been achieved in the current study, as both pectin and PVA offer unique advantages for probiotic encapsulation, including biocompatibility, controlled release capability and process ability. However, EE% depends on various factors, such as the specific requirements of the probiotics, the desired release profile and the targeted application. This fact has also been supported by Ma et al. [3], who observed that probiotics were successfully encapsulated in gum Arabic nanofibers, owing to their compact structure. Similar results were observed by Simonič et al. [16], who observed that probiotics were successfully loaded in sodium alginate and polyethylene oxide nanofibers.

### 3.2. Characterization of PEC/PVA Nanofibers

#### 3.2.1. Zeta Potential (ζ)

Zeta potential represents the electrical potential at the interface between a particle or a surface and the surrounding liquid medium. It quantifies the magnitude and polarity of the charge present on the surface of the particle or material. Zeta potential is an important parameter to determine in nanofibers because it provides insights into their surface charge and stability [17]. Nanofibers are typically composed of fine fibers with high surface-area-to-volume ratios, and their surface properties can significantly influence their behavior and performance in various applications [18]. In colloidal systems, such as nanofibers, zeta potential is primarily influenced by the ionization or adsorption of charged species at the surface. These charged species can include functional groups, ions from the surrounding medium or adsorbed surfactant molecules [19]. The results regarding the zeta potential of the blank (P_0_) and probiotic-loaded (P_1_) PEC/PVA nanofibers have been listed in Table 1. The results indicated that the blank (P_0_) and probiotic-loaded (P_1_) nanofibers showed negative values. It has been stated by Coelho Braga de Carvalho et al. [20] that zeta potential can occur as a negative value for several reasons, such as the fact that nanofibers have functional groups on their surfaces that can become ionized in an aqueous medium as these groups have a higher electron affinity, acquiring a negative charge, resulting in a negative zeta potential. Therefore, charged species, like ions, can adsorb onto the surface, contributing to a negative zeta potential. Thus, the zeta potential of a material such as nanofibers can be pH-dependent; nanofibers with ionized functional groups may acquire a negative zeta potential at certain pH values. The mean value for zeta potential was observed to be −7.55 mV and −10.22 mV for the P_0_ and P_1_ nanofibers, respectively. The mean results show significant (*p* < 0.05) differences for zeta potential. The current findings reveal that the PEC/PVA nanofibers loaded with probiotics (P_1_) showed lower zeta potential compared to the blank PEC/PVA nanofibers (P_0_). Thus, the decrease in zeta potential from −7.55 mV (P_0_) to −10.22 mV (P_1_) indicated that the probiotics were successfully encapsulated within the nanofibers of PEC/PVA. Ceylan et al. [21] also observed that probiotic bacteria were successfully encapsulated within poly-vinyl alcohol and sodium alginate nanofibers when the particle size was reduced from −6.29 mV to −7.74 mV. The particle size of nanofibers can affect their zeta potential. Smaller particles tend to have higher surface-area-to-volume ratios, meaning they have more exposed surface functional groups that can contribute to higher or lower zeta potentials. However, the formula in Section 2.6.1 is the preferred formula used for the calculation of zeta potential.

#### 3.2.2. Moisture Content (%)

Moisture analysis was conducted for both the blank (P_0_) and probiotic-loaded (P_1_) PEC/PVA nanofibers. Determining the moisture content in nanofibers is important for maintaining fiber stability, optimizing processing conditions, controlling material performance and ensuring storage stability [22]. Thus, moisture content may enhance the quality and functionality of nanofiber-based materials in various applications, such as food and biological systems. The results regarding moisture content are presented in Table 1. The mean moisture (%) for the blank PEC/PVA nanofibers (P_0_) was observed to be 14.11%, whereas the moisture content was increased in the probiotic-loaded PEC/PVA nanofibers (P_1_), at 14.27%. The increased moisture content observed in the probiotic-encapsulated nanofibers in the current study can be attributed to several factors such as the fact that the encapsulation matrix, pectin and PVA have efficient water retention properties, which may increase surface area and reduce moisture loss. These factors collectively contribute to the higher moisture content, which can be beneficial for the viability and functionality of the encapsulated probiotic cells [23]. Similar findings were observed by Sani et al. [24], who observed that after the encapsulation of sensitive compounds in apple peel pectin, the water holding capacity of the biofilm increased, which led to an increase in the targeted delivery of the bioactive compounds.

#### 3.2.3. Thickness

The thickness of nanofibers is a vital as it directly impacts the performance, functionality, mechanical properties, transport phenomena, fabrication process and application-specific requirements of nanofiber-based materials [25]. The results for the thickness of the PEC/PVA nanofibers, both blank (P_0_) and probiotic-loaded (P_1_), are listed in Table 1. The mean results obtained show significant (*p* < 0.05) differences for both blank and probiotic-loaded nanofibers. The average thickness for P_0_ (blank) was recorded as 0.127 mm, whereas for P_1_ (probiotic-loaded), the average thickness was recorded as 0.137 mm. A slight increase in the thickness of nanofibers has been affiliated with the probiotic encapsulation, as Ajalloueian et al. [26] reported that the thickness and diameter of pullulan electrospun nanofibers increased after probiotic encapsulation.

#### 3.2.4. Mechanical Properties (Tensile Strength and Elongation at Break)

The barrier properties of both the blank (P_0_) and probiotic-loaded (P_1_) PEC/PVA nanofibers in terms of tensile strength (TS) and elongation at break (%) were determined. Tensile strength refers to the maximum stress a material can withstand before it breaks or fails under tension. It is a measure of the material’s resistance to being pulled apart, whereas elongation at break (EAB) is a measure of the material’s ability to stretch or deform before it eventually fractures. It indicates the ductility or flexibility of a material. Nanofibers with high elongation at break can undergo substantial deformation without breaking, making them suitable for applications where flexibility and resilience are required [27]. The mean results are presented in Table 1. The tensile strength increased from the blank to probiotic-loaded nanofibers from 13.35 Mpa to 19.45 Mpa, respectively, which is a statistically significant (*p* < 0.05) difference. These results indicate that the tensile strength of the nanofibers significantly increased after the encapsulation of probiotics. Likewise, the mean results for EAB show a significant increase from the blank to probiotic-loaded nanofibers from 22.05% to 36.98%. The mechanical properties of the nanofibers increased after the encapsulation of probiotics. This result is supported by the findings of Duman & Karadag [28], who observed that the TS and EAB of nanofibers improved after the encapsulation of insulin. Increased barrier properties of nanofibers were also observed by Lin et al. [29], who stated that moringa-oil-loaded nanoparticles enhanced the tensile strength and elongation at break of gelatin nanofibers.

#### 3.2.5. Scanning Electron Microscopy (SEM)

**Figure 1:**
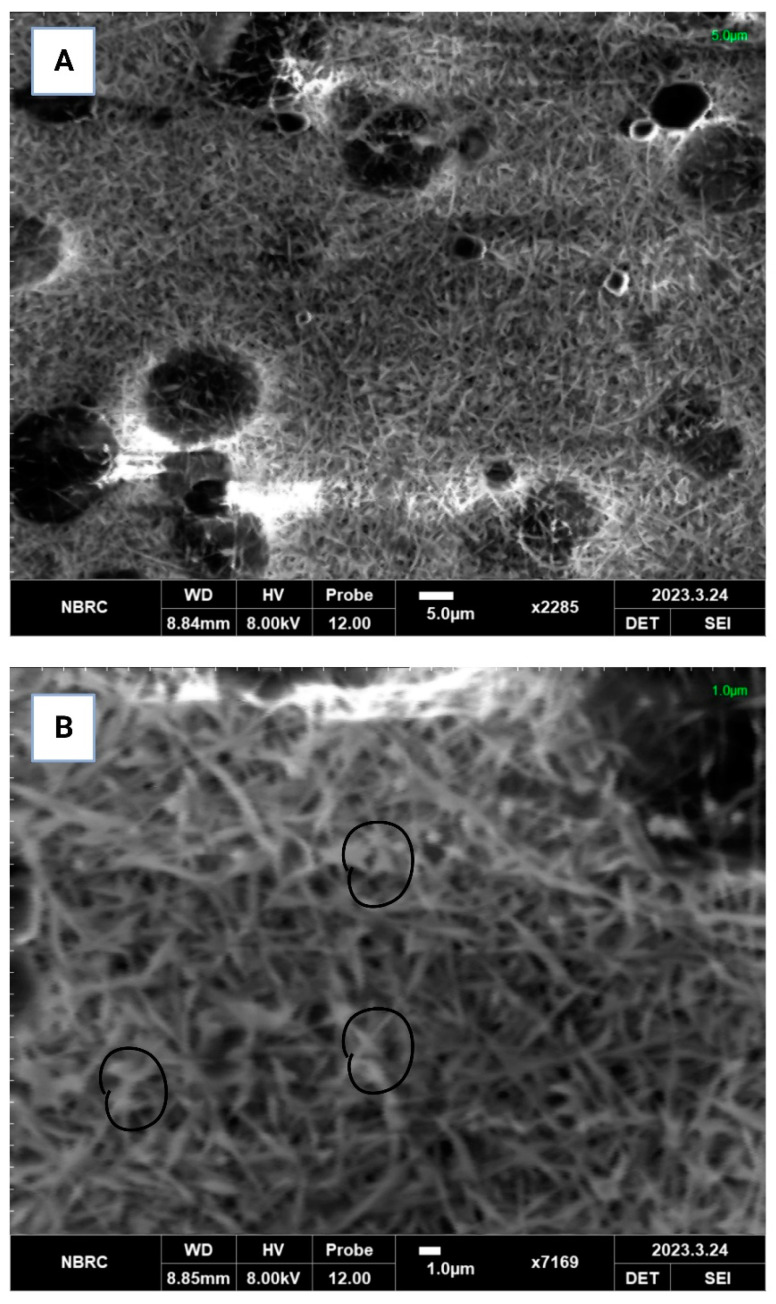
Scanning electron microscopy (SEM) observations of PEC/PVA nanofibers: (**A**) blank PEC/PVA, (**B**) probiotic-loaded PEC/PVA.

SEM micrographs were obtained to characterize the morphology, structure, surface properties and overall quality of the PEC/PVA nanofibers. Figure 1 shows that *L. acidophilus* was encapsulated in the PEC/PVA nanofibers. The micrographs show that the probiotics were randomly distributed among the nanofibers. The prominence of the probiotics in the SEM micrographs suggests the probiotics’ ability to establish and thrive within the fiber environment. This could be attributed to their capacity to adhere to fiber surfaces or potentially colonize fiber structures, thus leading to their visibility in the SEM images. The presence of probiotics alongside fine and thin fibers may imply a beneficial interaction between the encapsulant and coating matrix [30]. In this context, the proximity of the probiotics to fine and thin fibers might indicate a potential symbiotic relationship, where the fibers provide a conducive environment for the probiotics to exert their positive effects. The appearance of fine and thin fibers in the SEM micrographs could suggest the high quality the of fibers. Fibers that are fine and thin often exhibit desirable characteristics, such as increased surface area, improved texture and enhanced functionality. However, in the phenomenon of electrospinning, the electrostatic forces created by the electric field overcome the surface tension of the liquid solvent in the PEC/PVA solution. As a result, the solvent rapidly volatilizes or evaporates, leaving behind a polymer-rich solution. The charged *L. acidophilus* cells, being attracted to the jet, become entrapped within the rapidly solidifying polymer nanofibers. The polymer solution solidifies quickly upon solvent evaporation, trapping the cells within the nanofiber matrix. Similar phenomena have been found in various studies [31,32,33].

#### 3.2.6. Fourier Transform Infrared Spectrometry (FTIR)

**Figure 2:**
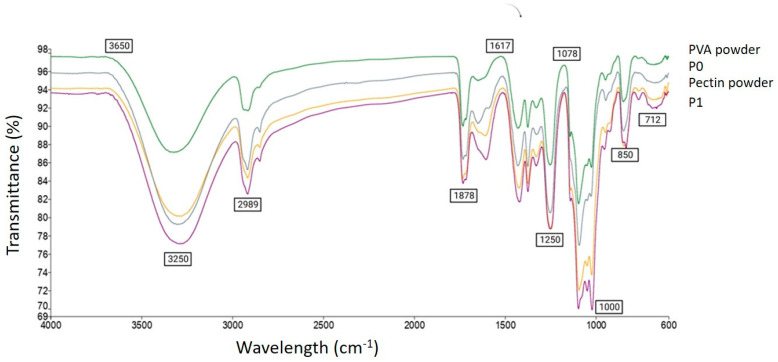
FTIR spectra of PEC powder, PVA powder, PEC/PVA nanofibers.

Figure 2 shows the FTIR spectra of the PVA powder, PEC powder and PVA/PEC nanofibers, both blank (P_0_) and probiotic-loaded (P_1_). In the PVA powder, the peak at 712 cm^−1^ indicates the presence of CH_2_ rocking vibrations, which are mainly characteristic of PVA. Pectin is a complex polysaccharide, and its FTIR spectrum exhibits various peaks related to different functional groups. The peak at 850 cm^−1^ is attributed to C-C stretching vibrations or the presence of PVA side chains. The peaks at 1000 cm^−1^ and 1078 cm^−1^ correspond to C-O stretching vibrations, which are typical for PVA as well as pectin. The peak at 1250 cm^−1^ indicates the presence of C-O-C asymmetric stretching vibrations in PVA. The peak at 1617 cm^−1^ is attribute to the presence of C=O stretching vibrations in PVA, suggesting the presence of carbonyl groups, whereas in the PEC powder, the peak at 1617 cm^−1^ indicates the presence of carboxylate groups (-COO-), which are characteristic of pectin. The peaks at 1878 cm^−1^ and 2989 cm^−1^ indicate O-H stretching vibrations, which are characteristic of PEC. The peak at 3250 cm^−1^ corresponds to O-H stretching vibrations, suggesting the presence of hydroxyl groups in PVA, and the peak at 3250 cm^−1^ corresponds to O-H stretching vibrations, indicating the presence of hydroxyl groups in pectin. The peaks observed for PVA and pectin (P_0_) in the individual spectra are expected to appear in the combined solution as well. The relative intensities of these peaks depend on the concentration and ratio of PVA and pectin in the solution. However, in the probiotic-loaded nanofibers (P_1_), the presence of probiotics in the combined solution introduced additional peaks and modified the intensities of existing peaks in the spectrum. Specific changes can be observed in the spectra, which indicates the interaction between the probiotics and the PVA–pectin matrix. These changes are related to new functional groups or alterations in the molecular structure of the components. Various studies have observed similar alterations of peaks that indicated the interaction of compounds with each other [3,9,34].

#### 3.2.7. Thermogravimetric Analysis (TGA)

**Figure 3:**
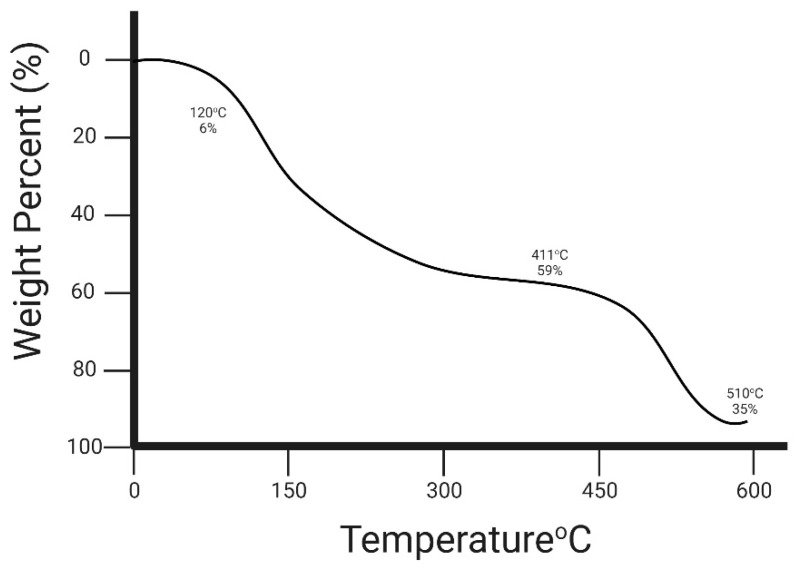
Weight degradation (%) of PEC/PVA nanofiber at high temperature.

The thermal stability of the PEC/PVA nanofibers was assessed in the current study, and the results are shown in Figure 3, as TGA can help to determine the onset of decomposition, identify the presence of impurities and estimate the thermal stability of nanofibers. By analyzing the weight loss patterns at different temperatures, studies gain insights into the chemical composition, thermal stability and degradation properties of nanofiber materials. The thermographs of both the blank and probiotic-loaded PEC/PVA nanofibers indicated weight loss observed during TGA, which may indicate the presence of volatile components, such as solvents or residual organic materials, present in the nanofibers. The thermal degradation with the increase in temperature was observed in three phases. The first degradation of the blank PEC/PVA nanofibers was observed from 0 to 120 °C and 6% weight loss was observed. The second degradation phase was observed between 160 °C and 411 °C, and a significant weight loss of 59% was observed. This massive weight loss was observed due to the thermal degradation of galacturonic acid chains in PEC [35]. However, at 411 °C to 510 °C, 35% weight loss was observed as at this stage, the degradation mainly occurred due to PVA concentration [36]. However, the increased degradation temperature indicates the interaction between PEC and PVA.

### 3.3. In Vitro Testing

#### 3.3.1. Viability under Simulated Gastric Conditions

**Figure 4:**
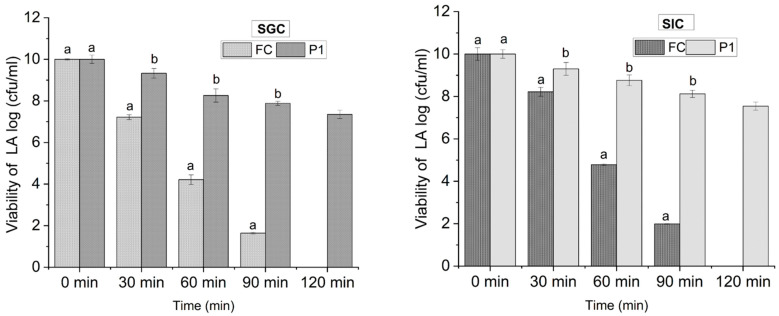
Viability of LA under simulated gastric and intestinal conditions. FCs = free cells of probiotics, P_1_ = probiotic-loaded PEC/PVA nanofibers, SGCs = simulated gastric conditions, SICs = simulated intestinal conditions. Reported values are mean ± SD (n = 3) and small letters (a,b) over error bars represent significant differences (at *p* < 0.05) between treatments using one-way ANOVA and Duncan’s multiple range test.

The human stomach is a highly acidic environment with a low pH that can range from 1 to 3, which poses a significant challenge to the survival of probiotics, as it can lead to a decrease in their viability [37]. By subjecting encapsulated probiotics to simulated gastric conditions, the viability of probiotics under the harsh conditions of the stomach can be assessed to evaluate the survival rate of the probiotics more realistically. The results regarding the viability of free cells (FCs) and cells encapsulated in PEC/PVA nanofibers (P_1_) are shown in Figure 4. In the simulated gastric fluid at pH 2 (acidic condition), the viable count for free cells was recorded as 7.22 log (cfu/mL) at 30 min. However, a reduction to 1.64 log (cfu/mL) was observed at 90 min, whereas the viability of FCs was undetected at 120 min. In contrast to this, the viability of encapsulated probiotics in the PEC/PVA nanofibers (P_1_) was recorded as 9.33 log (cfu/mL) at 30 min, which was later reduced to 7.35 log (cfu/mL) at 120 min. These findings reveal that the viability increased from 0 log (FCs) to 7.35 log (cfu/mL) (P_1_) among free cells and encapsulated cells at 120 min, respectively. The results of the current study are supported by Atraki & Azizkhani [2], who observed that the viability and survival rate of probiotic strains were enhanced and retained up to 81% after encapsulation in corn starch and sodium alginate nanofibers.

#### 3.3.2. Viability under Simulated Intestinal Conditions

Similarly, both free cells (FCs) and cells encapsulated in nanofibers (P_1_) were subjected to simulated intestinal conditions (SICs). The results are presented in Figure 4. The results reveal that on exposure to SICs at pH 6.8 (slightly acidic), the viability count for FCs was recorded as 8.22 Log at 30 min and 1.99 log (cfu/mL) at 90 min, whereas the viability of FCs was not detected at 120 min. In contrast, it was found that probiotic cells encapsulated in the PEC/PVA nanofibers (P_1_) were more viable than free cells, with a maximum viability of 9.31 at 30 min and a minimum viability of 7.54 at 120 min, though cells were still viable. The findings in Figure 4 also suggest that the viability of both free cells and encapsulated decreased drastically in SGCs (pH 2) and cells’ viability was decreased to a lesser extent in SICs (pH 6.8) as compared to SGCs (pH 2). These findings are supported by the findings of Yu et al. [38], who observed increased viability of probiotics on exposure to intestinal stock solution up to 7 log (cfu/mL). However, the combination of low pH, digestive enzymes, mechanical agitation and the presence of bile salts in the gastric environment collectively contribute to the decreased viability of encapsulated and free cells of probiotics compared to the more favorable conditions present in the intestines [39].

## 4. Conclusions

Nanofibers remain the prime choice for the encapsulation of probiotics, as they are not only a biodegradable material but also an excellent carrier for probiotics. This research concluded that pectin is a beneficial choice for enhancing the viability of probiotics under SGCs and SICs. Additionally, the addition of polyvinyl alcohol (PVA) improves the barrier properties of nanofibers. However, equal ratios of pectin and PVA (10:10) demonstrate excellent spinnability in electrospinning. This combination proves to be a promising approach for increasing the viability and stability under stressed conditions. However, future work could investigate the modification of pectin by means of esterification or using various types of pectin with diverse molecular weights and average chain lengths, which may enhance the survivability of probiotics. The development of new nanofibers opens a new window for the increased survival of probiotics, which may be helpful for the development of new functional foods.

## Figures and Tables

**Table 1:** Zeta potential, moisture, encapsulation efficiency and mechanical properties of nanofibers.

Parameter	P_0_ (*)	P_1_ (*)
Zeta potential (mV)	−7.55 ± 0.08 ^a^	−10.22 ±0.01 ^a^
Moisture content (%)	14.10 ± 0.06 ^a^	14.27 ± 0.05 ^a^
Encapsulation efficiency (%)	---	82.90%
Mechanical properties		
Thickness (mm)	0.128 ± 0.05 ^a^	0.137 ± 0.01 ^b^
Tensile strength (MPa)	13.35 ± 0.04 ^a^	19.45 ± 0.01 ^b^
Elongation at break (%)	22.05 ± 0.05 ^a^	36.98 ± 0.01 ^b^

* P_0_ = PEC/PVA blank nanofibers, * P_1_ = probiotic-loaded PEC/PVA nanofibers. Values are presented as mean ± SD (n = 3) while small letters (a,b) show significant differences between treatments using one-way ANOVA and Duncan’s multiple range test at 5% of level of significance.

## Data Availability

The data used to support the findings of this study can be made available by the corresponding author upon request.
